# Successful Management of Early Failure (One Month) of a Standard Maxillary First Molar Implant After Sinus Lifting Using a Short Implant: A Case Report

**DOI:** 10.7759/cureus.104711

**Published:** 2026-03-05

**Authors:** Maxime Lavigogne, Laurence Senoussi, Georges Khoury, Osama Alhassan, Alp Alantar

**Affiliations:** 1 Oral Surgery, Hôpital Max Fourestier, Nanterre, FRA; 2 Dentistry, Hôpital de Nanterre, Nanterre, FRA; 3 Implantology, Hôpital de Nanterre, Nanterre, FRA

**Keywords:** alternative treatment, bone loss, failure, periodontal status, short implant, sinus lift, standard implant

## Abstract

The use of short implants (5 mm diameter × 6 mm length) has increased in recent years, and they have mostly been indicated in the posterior maxillary areas in cases of insufficient bone height, as an alternative to sinus lift procedures. Short implants have rarely been proposed in cases of early failure (less than one month) of maxillary first molar standard implants placed after sinus lifting.

We are reporting a 64-year-old female smoker who was referred for consultation concerning the extraction and implantation of tooth #16. The periodontal preparation and smoking cessation were followed by a sinus lifting. After a 10-month post-graft period, a standard implant (4.7 × 11.5 mm; Zimmer Biomet®, Warsaw, IN, USA) was placed. One month postoperatively, early failure occurred due to failure in osseointegration, and the implant was removed; three months later, a short implant (5 mm diameter × 6 mm length) was placed. The second stage of surgery was carried out at six months postoperatively. After four weeks, a crown was set. The control at 13 months showed a bone-integrated implant without lateral bone loss. The main risk factors associated with implant failure are low bone density, sinus grafting, and chronic smoking. In this case report, the association of these three risk factors led to standard implant failure. A short implant was proposed in order to avoid a new bone graft. A 2:1 crown-to-implant ratio has no impact on marginal bone loss or implant survival. In conclusion, the short implant (5 mm diameter × 6 mm length) may be a minimally invasive alternative in cases of early loss (less than one month) of a standard implant placed after sinus lifting without any additional bone grafting procedure. This data must be confirmed with a cohort study.

## Introduction

In cases of early implant failure after sinus augmentation, the conventional approach usually involves a new grafting procedure, followed by delayed implant placement after several months of healing [1-3]. Such management increases morbidity, treatment time, and cost, and may negatively affect patient comfort and satisfaction. In this context, the use of short implants could represent a valuable alternative by avoiding additional grafting procedures and simplifying treatment, while maintaining high survival rates [4].

The use of short implants (6 mm in length) has increased in recent years and has been mostly indicated in the posterior maxillary areas in cases of insufficient bone height, as an alternative to sinus lift [5,6]. To our knowledge, short implants have never been proposed in cases of early failure (less than one month) of standard implants placed after sinus lifting. We report a case of early failure (one month) of a standard maxillary implant placed after lateral approach sinus grafting, which was successfully replaced by a short implant. The advantages of such an alternative to a new bone graft are discussed.

## Case presentation

**Figure 1:**
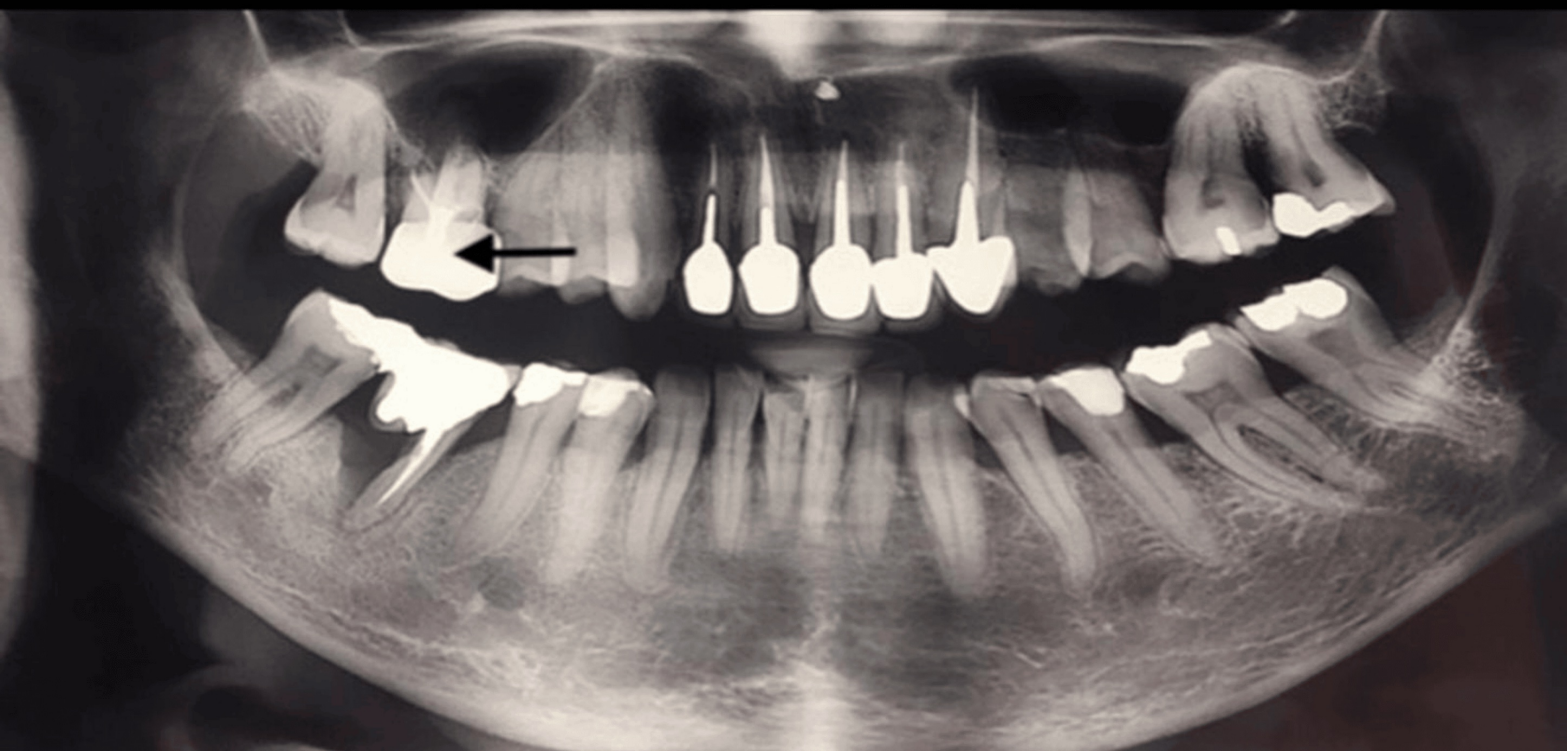
Orthopantomographic view Mobility and 50% radiographic bone loss of tooth #16 (arrow), indicating its extraction.

A 64-year-old female smoker (54 pack-years), with a history of hemorrhagic rectocolitis, was referred for the extraction and implantation of the mobile tooth #16 (Figure 1).

**Figure 2:**
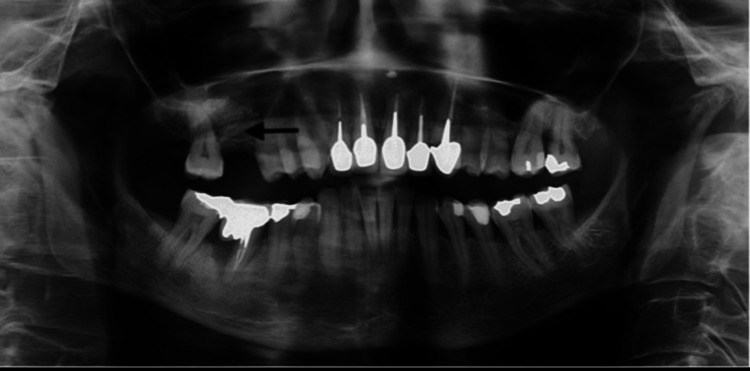
X-ray control after sinus grafting X-ray control after right maxillary lateral approach sinus grafting (β TCP/HA) (arrow). β-TCP: beta-tricalcium phosphate

The residual bone height of the 4/5 mm sinus floor indicated a sinus lifting procedure. The extra-oral examination was normal, while the intra-oral examination revealed localized stage III periodontitis (alveolar bone loss >33%) with an asymptomatic vertical intra-osseous lesion following a root amputation on pillar 46 of the bridge placed about 13 years ago. Periodontal support included initial therapy consisting of three sessions of scaling, root planing, and pocket rinse with 10% betadine mixed with oxygenated water 10 vol 3%, especially of #46 (12 months post-treatment probing depth: 4 mm), along with cessation of smoking. A lateral approach sinus grafting (beta-tricalcium phosphate (β-TCP/HA), collagen membrane) (Figure 2), under local anesthesia (articaine 2% with adrenaline 1:100,000), and perioperative and postoperative 15-day antibiotic prophylaxis (penicillin 500 mg/clavulanic acid 62.5 mg combination, 2 g/day) followed the etiological periodontal treatment. Pain was controlled by paracetamol/codeine prescription. 

**Figure 3:**
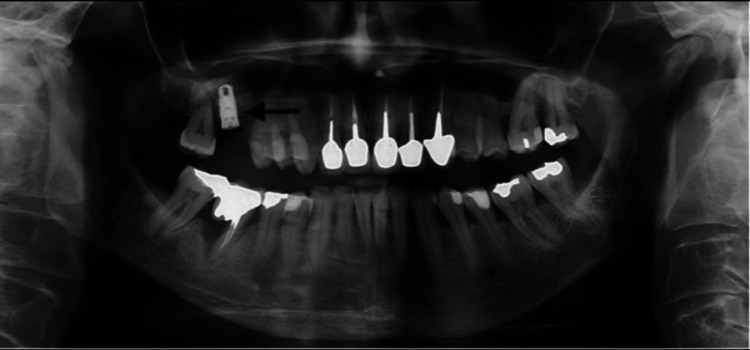
View of the implant placed six months after sinus lifting A 4.7 x 11.5 mm implant (Zimmer® TSV) (arrow) placed six months post-sinus lifting.

At 10 months post-graft, a standard 4.7 x 11.5 mm Zimmer® TSV implant (Zimmer Biomet®, Warsaw, IN, USA) was placed on healthy periodontal tissue (Figure 3).

One month after the surgical procedure, the implant was removed because of failure of osteointegration. At the time of failure, only a residual pocket (5 mm) was noticed on tooth #46. The patient reported that she had smoked again after surgery. The implant was removed under antibiotic therapy, with curettage and irrigation using a betadine 10% solution/oxygenated water 10 vol 3% mixture, and a fibrinous hemostatic sponge was applied. Resorbable sutures without tension completed the procedure. The integrity of the implant site was checked during the periodontal curettage.

**Figure 4:**
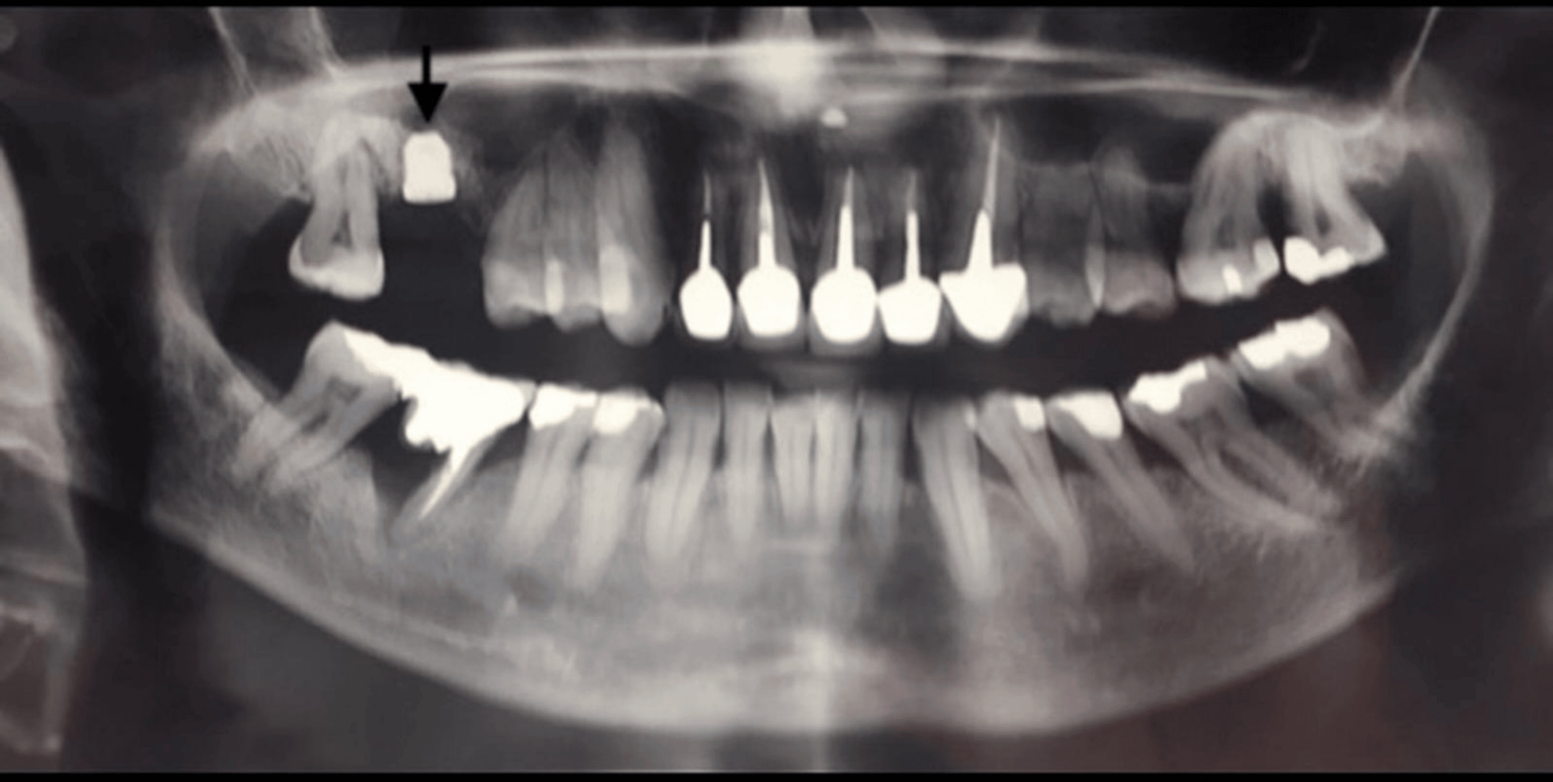
View of short implant placed after three months Three months after implant loss and definitive permanent smoking cessation, a short implant (Zimmer Biomet T3®, 5 mm (D) x 6 mm (L)) was placed (arrow) at bone level.

Three months after surgery and definitive permanent smoking cessation, a short implant (Zimmer Biomet T3®, 5 mm diameter × 6 mm length) was placed at bone level (Figure 4) under local anesthesia (articaine 2% with adrenaline, 1:100,000).

A 15-day antibiotic prophylaxis (penicillin and clavulanic acid combination, 2 g/day) was prescribed. Pain management was achieved using a combination of paracetamol (400 mg) and codeine (20 mg).

The second-stage surgery was performed six months later. As the gingival width was sufficient, flapless implant surgery was indicated in order to minimize marginal bone loss and ensure better patient comfort [3]. An incision was made with a 6 mm diameter biopsy punch (Stiefel®, Triangle Park, NC, USA). A 5.6 x 6.0 mm conical healing abutment (WTH56) was placed. After four weeks of peri-implant gingival healing, a screw-retained metal-ceramic crown was made with a 2:1 crown-to-implant ratio.

**Figure 5:**
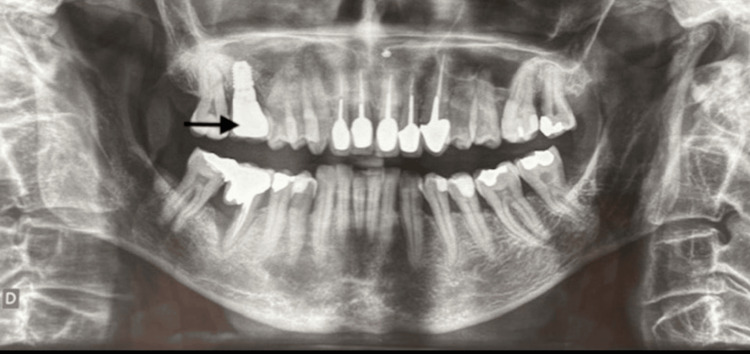
View at 13-month post-prosthetic follow-up (arrow)

Canine-guided occlusion was established in lateral movements to prevent occlusal interference from contact between the supra-implant crown and mandibular teeth. The 13-month check shows an osseointegrated implant without marginal bone loss or pathologic pocket depth (3 mm) (Figure 5).

Biannual periodontal maintenance, including scaling, root planing, and rinses with betadine 10% solution/oxygenated water 10 vol 3%, was performed, especially around the 46/47 bridge.

**Figure 6:**
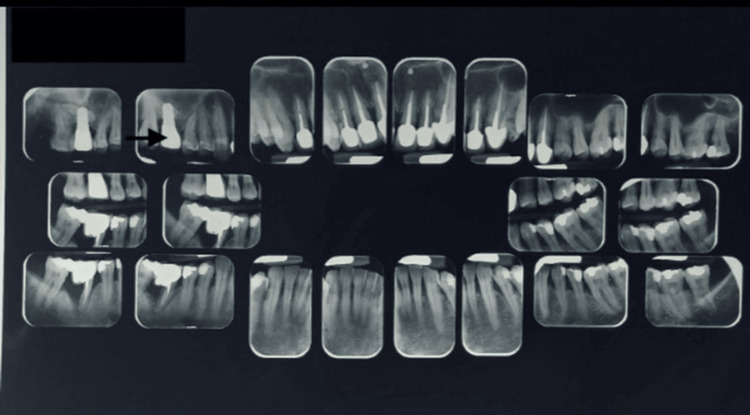
View at 27-month follow-up: full-mouth long-cone periapical radiographic survey (arrow), following biannual scaling and root planing

A 27-month follow-up after short implant placement was conducted (Figure 6); clinical examination and intraoral radiographic view revealed a bone-level implant.

**Figure 7:**
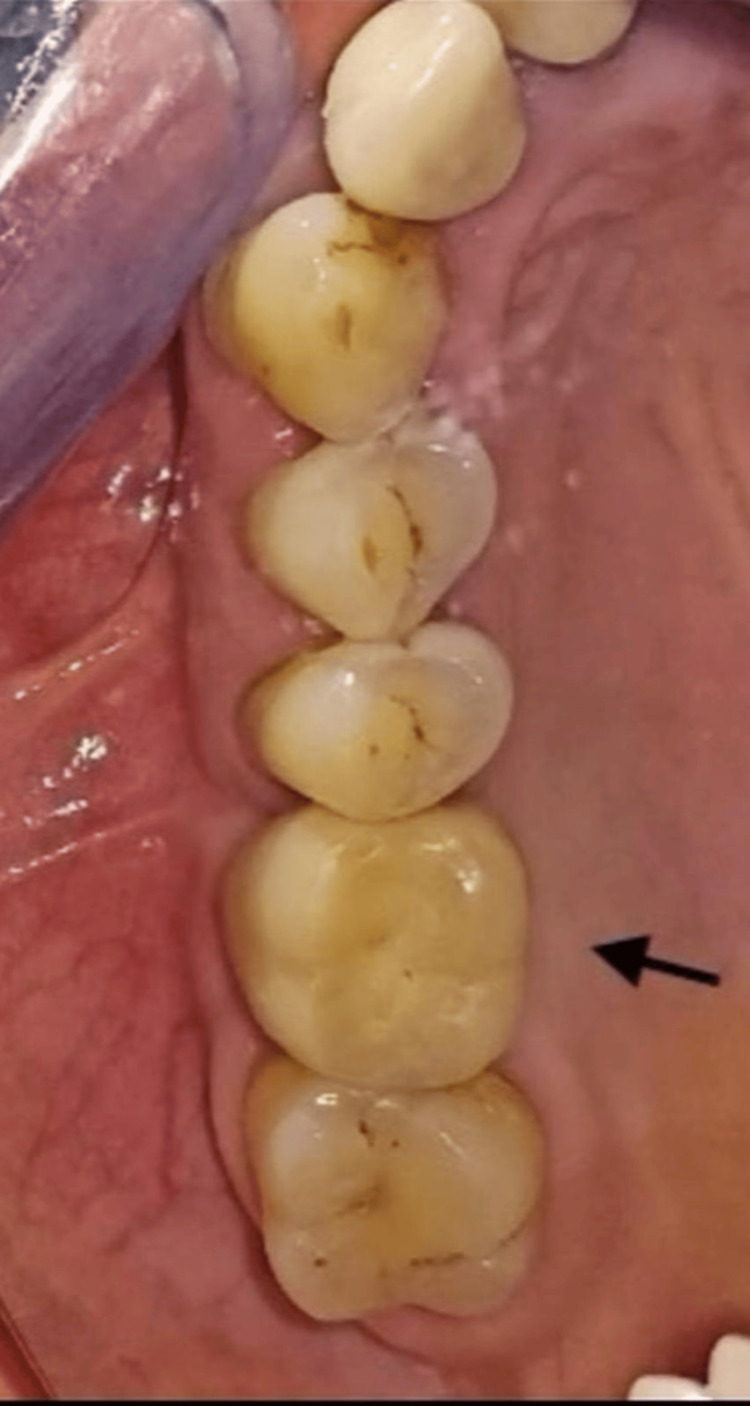
Clinical view Screw-retained metal-ceramic crown (#16) (arrow), at 27-month post-prosthetic loading follow-up.

No marginal bone resorption, no mobility, no bleeding, and a 5 mm pocket depth at teeth #46/47. Clinically, the screw-retained metal-ceramic crown shows no mobility (Figure 7).

## Discussion

The main risk factors associated with the loss of standard implants are type III/IV bone density, pre-implant sinus grafting, and chronic smoking [4]. Only periodontal pockets adjacent to the implant are implicated in peri-implantitis; the opposite maxillary sites (as in this case, with tooth #46) and contralateral sites have no impact on the occurrence of peri-implantitis [5]. In this case report, the early failure of the implant was strongly related to the combination of two risk factors: chronic tobacco use and poor bone graft quality. The position of the implant, very close to the pronounced angular bone resorption in the mesial area of tooth #17, may also have contributed to the failure. Following re-implantation, the angulation was corrected, and the implant was placed farther from the resorptive region of tooth #17, which may likewise have been a key factor in the success of the second implantation. Short implants help avoid complex surgeries; they reduce surgical time, pain, bleeding, and infectious complications [6]. They cause fewer biological complications than standard implants [7]. The five-year survival rate of posterior short implants ranges from 86.7% to 100%; the prosthetic survival rate ranges from 90% to 100% [8].

Compared to standard implants placed after sinus lifting, short implants show no significant difference in survival rate (p = 0.08) or marginal bone loss (p = 0.08). They have fewer biological complications (p < 0.00001), but more prosthetic complications than standard implants (p = 0.01). A 2:1 crown-to-implant ratio does not impact marginal bone loss, prosthetic instability, or implant survival rate [9,10], and their use can be considered favorable and fully justified.

According to Toledano et al. [5], at three years, short implants showed a significantly higher mean implant stability quotient than the standard-length group. Nevertheless, implant stability measurements (mesiodistal and buccolingual) across the follow-ups showed no significant difference between the two treatment groups, confirming that the application of sinus elevation did not influence implant stability, regardless of implant length.

The primary advantage of placing a short implant following an early failure (less than one month) of a standard implant after sinus lift surgery lies in its minimally invasive nature. The short implant serves as a salvage option, avoiding the need for an additional bone grafting procedure. As early implant failure (less than one month) does not lead to bone loss, the placement of a short implant is feasible following a routine panoramic radiograph. This advantage is cost-effective for the patient. In cases of inflammatory or painful symptoms, a new cone beam computed tomography (CBCT) is recommended. In late failure cases, new CBCT imaging is systematically indicated.

## Conclusions

The short implant (5 mm diameter × 6 mm length) may be a minimally invasive alternative in case of early (less than one month) loss of a maxillary first molar standard implant after sinus lifting; it eliminates the need for a new bone graft and CBCT, and improves patient comfort. A five-year cohort study will be required to confirm this assumption.
